# Donor/Recipient Delta Age: A Possible Risk for Arterial Stenosis in Renal Transplantation

**DOI:** 10.1155/2015/512929

**Published:** 2015-12-31

**Authors:** Giovanni Pallotti, Gabriele Donati, Irene Capelli, Olga Baraldi, Giorgia Comai, Patrizia Agati, Michele Nichelatti, Giuseppe Cianciolo, Gaetano La Manna

**Affiliations:** ^1^Faculty of Medicine and Surgery, Department of Physics, University of Bologna, Via Berti Pichat 6/2, 40127 Bologna, Italy; ^2^Nephrology Dialysis and Renal Transplantation Unit, S. Orsola University Hospital, Via Massarenti 9, 40138 Bologna, Italy; ^3^Department of Statistical Sciences, University of Bologna, Via Belle Arti 41, 40126 Bologna, Italy; ^4^Service of Biostatistics, Niguarda Cà Granda Hospital, Piazza Ospedale Maggiore 3, 20162 Milan, Italy

## Abstract

Different arterial wall properties can significantly increase the risk of blood turbulent fluxes leading to complications such as atherosclerosis. Since the mechanical properties of arterial vessels are influenced by age, we investigated, in a retrospective study, the effects on renal artery stenosis of an age difference >15 years between donor and recipient in a cohort of 164 patients undergoing renal transplantation between 1981 and 1991. The age difference between donor and recipient was ≤15 years in 87 patients (53.0%) (Group A) and >15 years in 77 patients (47.0%) (Group B, *p* = ns). None of the Group A patients developed an anastomotic arterial stenosis, whereas 8/77 Group B patients (10.4%) had an anastomotic arterial stenosis (*p* < 0.001). This study shows that an age difference >15 years is significantly linked to the risk of developing arterial stenosis after renal transplantation. Indeed, different wall properties can significantly increase the risk of generation of blood turbulent fluxes and involve, in the arterial vessels, the development of complications such as atherosclerosis.

## 1. Introduction

**Figure 1:**
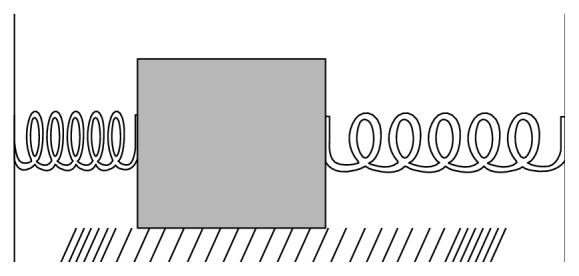
Coulombian resistance (modified from [5]).

Despite the great efforts carried out in recent years to ameliorate immunological [1] and nonimmunological [2] tolerance of renal allograft after transplantation, little is known about the role of the mechanical connection between the great arterial vessels of the donor and those of the recipient after transplantation. The theory of viscous-elastic media has been used to investigate the mechanical properties of the great vessels, analyzing the sphygmic wave [3] traveling into the structures of the arterial tree and studying the risk of blood turbulent flow inside the vessels. Maxwell, Moritz, and Anliker [4, 5] evaluated the elastic properties of the great arterial vessels and their wall impedance [6, 7] in living tissues of Alsatian dogs chosen because this cardiovascular system is very similar to the human system [4, 5]. They developed a method to generate and record small-amplitude waves (pressure, torsional, and axial) in exposed canine carotid artery [4, 5]. The expressions obtained showed the nonlinearity of the viscous-elasticity of the great arterial vessel tissue, due to Coulombian resistance (Figure 1). If any deformation occurs in a biological soft tissue, the recovery of the original condition is not given by a curve of equal shape but by Coulombian resistance to the deformation [8, 9]. Coulombian resistance consists in a small resistance when the tissue is dragged from the arriving sphygmic wave and in a high resistance when the tissue returns to the initial condition of strain: a vessel section spends less time to reach the maximum of its own diameter than it does to return. This is related to “hysteresis” due to viscoelasticity of the arterial wall. In particular, the behavior can be simulated by two springs acting as the wave crosses the vessel section, with a very low damping of the recovery of the original tissue size (Figure 1).

After organ transplantation, the first mechanical effects are observed in blood vessels and in blood itself when the arteries have been connected and the pressure wave starts being transmitted from the body into the new element present in the circulatory system. The most delicate site is the suture line. The clinical consequences of this event are a high shear stress on the patient's vascular wall, activation or acceleration of atherosclerotic processes, and the formation of vessel stenosis, significantly reducing blood flow to the graft [10–12].

## 2. Mechanical Properties of Great Vessels

From the results obtained by Moritz and Anliker [5], we see that phase velocities of the different types of waves can be considered constant in the interval of the frequencies considered. Note the values of the experimental velocities and attenuations given by the general relationship of different waves as shown in Table 1, where *V*_*r*_ is the radial velocity, *V*_*a*_ the axial wave velocity, and *V*_*t*_ the torsional wave velocity, while *α*_*r*_^2^ is the radial attenuation, *α*_*a*_^2^ the axial wave attenuation, and *α*_*t*_^2^ the torsional wave attenuation.

In this situation, the biological tissue shows the same mechanical properties of a Knopoff body [6, 10, 11], and therefore, it must be investigated using the partial differential equation:

(1) $$\begin{array}{c} \rho \frac{\partial^{2}\overset{-}{u}}{\partial t^{2}}=\mu_{E}\frac{\partial^{2}u}{\partial x^{2}}+\mu_{v}\frac{\partial^{3}\overset{-}{u}}{\partial {t\partial x}^{2}}, \end{array}$$

where *ρ* is the density, *t* the time, *x* the axial coordinate, *μ*_*E*_ the elastic constant, and *μ*_*v*_ a second constant with the dimension of a viscosity, while

(2) $$\begin{array}{c} \overset{-}{u}=u+u_{p} \end{array}$$

is the total displacement, given by the sum of the recoverable displacement *u* and the permanent static displacement *u*_*p*_.

**Figure 2:**
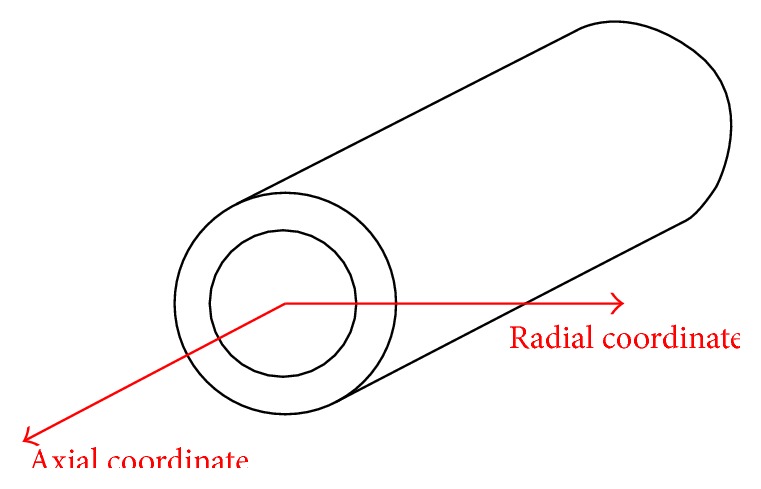
Schematic representation of the variables considered.

The solution of Knopoff differential equation (1) is

(3) $$\begin{array}{c} u=A\exp\left(\;-\frac{\alpha^{2}x}{\lambda }\;\right)\sin\left(\;\omega t-kx\;\right), \end{array}$$

in which *α* is a constant, *λ* the wavelength, *ω* the pulsation, and *k* the wave number. In turn, the theoretical radial *V*_*r*_ and axial *V*_*a*_ wave velocities (Figure 2) are, respectively, given by

(4) Vr=−μEρ1/2,

(5) Va=−λE+μEρ1/2,

*λ*_*E*_ being the volume compressibility.

To examine the transmission of an oscillation by an arterial wall, we must consider only the longitudinal element of the vessel, without taking blood flow into account because only the wall transmits such movements [13]. The expression of attenuation, in the two different cases of radial and axial waves, can be given on the basis of the constant coefficients *α*_*r*_^2^ and *α*_*a*_^2^, respectively, as follows:

(6) 4ηcρVr2xλ=αr2xλ,

(7) 2ζc+ΨcρVa2xλ=αa2xλ,

where *η*_*c*_, *ζ*_*c*_, and Ψ_*c*_ are all constants with the dimensions of the reciprocal of a stress, and therefore, from (6) and (7), and from their ratio

(8) $$\begin{array}{c} \varepsilon =\frac{2\eta_{c}}{\zeta_{c}+\Psi_{c}}\frac{V_{r}^{2}}{V_{a}^{2}}, \end{array}$$

we evolve that both *V*_*r*_ and *V*_*a*_ velocities must be constant.

Now we examine equations of the phase velocities obtained. From the theory of elasticity, we consider the Lamé constant [8] in agreement with the isotropy of the system.

For the radial wave, using Young's modulus *E* and the Poisson ratio *σ*, we can then write

(9) Vr=E2ρ1+σ1/2,Va=E1−σρ1+σ1−σ1/2

such that the ratio between radial and axial component is

(10) $$\begin{array}{c} \frac{V_{r}}{V_{a}}={\left(\;\frac{1-2\sigma }{2\left(1-\sigma \right)}\;\right)}^{1/2}. \end{array}$$

### 2.1. Numerical Considerations

Utilizing the experimental values obtained by Moritz and Anliker [5], for example, *V*_*r*_ = 1.1 × 10^3^ cm · s^−1^ and *V*_*a*_ = 3.0 × 10^3^ cm · s^−1^, we obtain a Poisson ratio *σ* = 0.422, while Young's modulus (equal for the axial and radial waves) and the volume compressibility, respectively, are *E* = 3.786 × 10^6^ dyne · cm^−2^ and *λ*_*E*_ = 7.220 × 10^6^ dyne · cm^−2^.

The bulk modulus of the artery is therefore *κ* = 8.125 × 10^6^ dyne · cm^−2^, so that *η*_*c*_ = 2.066 × 10^−7^ dyne · cm^−2^, and the model presents a characteristic Coulombian resistance.

## 3. Aim of the Study

**Figure 3:**
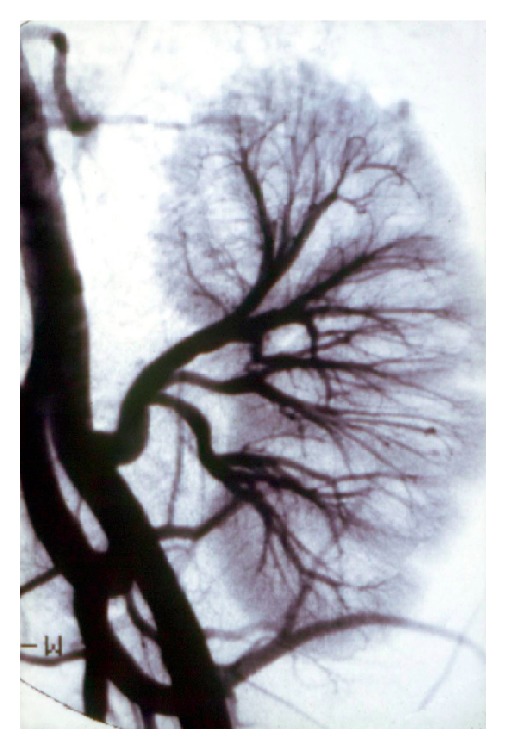
Normal arterial anastomosis in a kidney transplant recipient.

**Figure 4:**
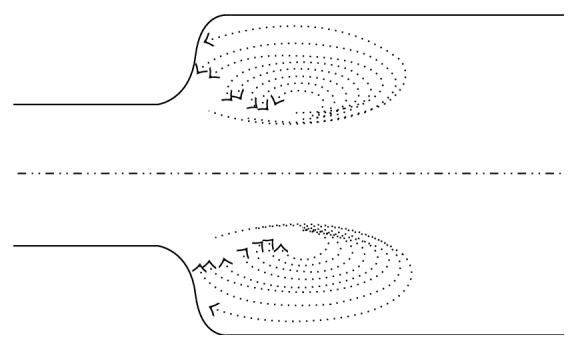
Laminar flow alterations depending on wall impedance and blood flow disturbances.

For an experimental validation of the mathematical relationship between the arterial wall impedentiometry and the blood flow disturbances represented by (1)–(3), the arterial connection occurring in renal transplant was considered. During renal transplantation, different arterial segments are connected in series (Figure 3). The different biomechanical impedance of the arterial segments of donor and recipient generates a discontinuity on the arterial wall and only part of the incident blood energy intensity can be transmitted across the anastomosis, whereas the remaining energy intensity is reflected by the system [13]. Therefore wall deformations are generated by the blood pressure shearing stress (Figure 4). The present study is a theoretical evaluation of the arterial wall elastic properties on the development of an anastomotic arterial stenosis in the transplanted organ. Since the mechanical properties of arterial vessels are influenced by age, we investigated the effects on renal artery stenosis of an age difference >15 years between donor and recipient in a cohort of 164 patients undergoing renal transplantation.

## 4. Patients and Methods

Among 352 patients who received kidney transplants at the Nephrology Dialysis and Renal Transplantation Unit of the S. Orsola University Hospital, Bologna, 164 patients were retrospectively evaluated by a mathematical elaboration of the patients' data. All the patients were on regular dialysis treatment. Patient characteristics are summarized in Table 2. Donors aged >60 years and pediatric donors were not considered. The cause of donor death was head trauma in all cases. The clinical examination and the history of the potential recipients were considered to assess their comorbidities and their eligibility for the waiting list for renal transplantation. As to the definition of ischemic cardiac injury, cerebral and peripheral arteriopathy, the methods reported in a previous study by our group, were followed [14]. The organ allocation consisted in clinical and immunological matching between the donor and recipient. The clinical matching included age and weight differences between donor and recipient; the immunological criteria considered the HLA class I and class II match and the subsequent crossmatching by means of the ELISA test between the donor T and B lymphocytes and the recipient blood serum [15]. No donors younger than 18 years were considered. The threshold of 15 years of age difference between the donor and the recipient was considered because, from the early 1990s, it emerged as one of the main concerns in relation to kidney graft survival for both living and cadaveric donation [16, 17]. The time period considered for the present study started in January 1981 and ended in December 1991 because from January 1992 each renal transplant recipient in our Centre was regularly matched with a donor aged ≤15 years. The surgical team was the same throughout the period considered and the same nephrological team carried out the posttransplant follow-up. The arterial anastomosis in the transplanted kidney was a terminolateral anastomosis between the renal artery of the donor and the common iliac artery or the external iliac artery of the recipient. This kind of surgical technique did not change during the period considered. The onset of renal artery stenosis was considered during a 12-month follow-up. Diagnosis of renal artery stenosis was established by means of selective renal arteriography. It was carried out in case of the following: (a) a new onset arterial hypertension resistant to the current multiple (≥3) antihypertensive treatment when the patient's mean diastolic blood pressure was 90 mmHg or more; (b) a bruit clearly heard over the transplanted kidney on three consecutive clinical visits or at the time of admission to the hospital [18]. The same radiological team independently interpreted the selective renal arteriography, and an arteriographic narrowing ≥50% was required as the minimum criterion for the definition of a stenotic lesion. The present criterion for arterial graft stenosis is in accordance with the recent findings of Hagen and Ghazanfar who excluded from their cohort of transplanted patients with kidney graft arterial stenosis those with a stenotic lesion < 50% [19, 20]. Surgical causes of renal artery stenosis were excluded from the study; clinically they were characterized by a sudden increase in blood pressure (mean diastolic BP ≥ 90 mmHg) in spite of antihypertensive therapy few days after renal transplantation: stenosis of the suture line, angulation stenosis, and single segmental stenosis due to surgical trauma were assessed by means of renal selective arteriography [12]. Between-group differences were analyzed by *χ* ^2^ test, Wilcoxon test, or Student's *t*-test when appropriate. A *p* value <0.05 was considered statistically significant.

## 5. Results

**Figure 5:**
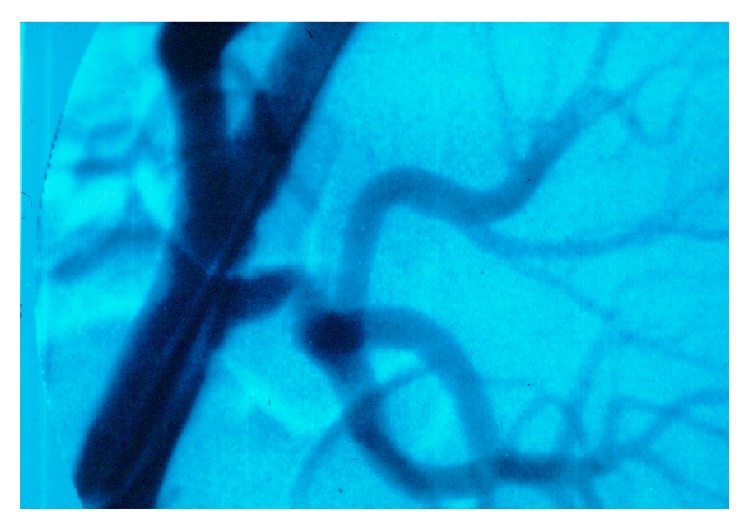
Arterial stenosis in a kidney transplant recipient (case 1).

**Figure 6 fig6:**
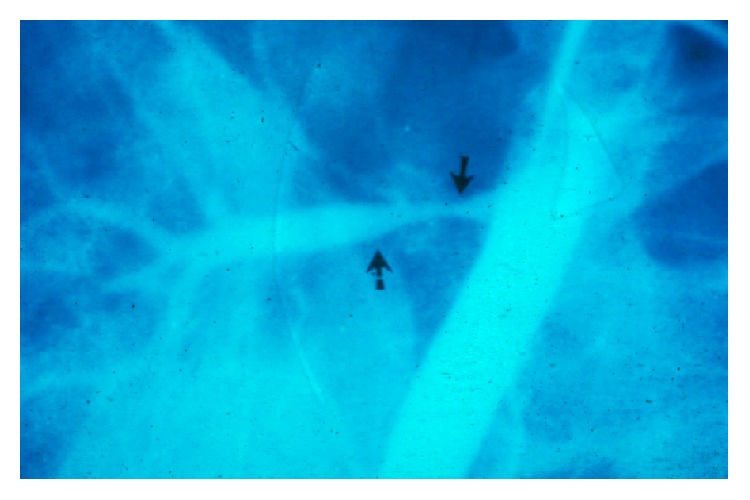
Arterial stenosis in a kidney transplant recipient (case 2).

**Figure 7 fig7:**
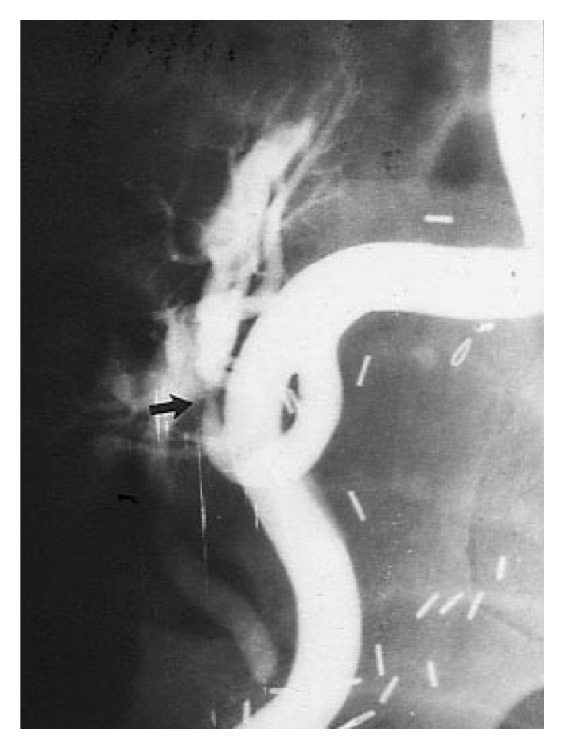
Arterial stenosis in a kidney transplant recipient (case 3).

**Figure 8 fig8:**
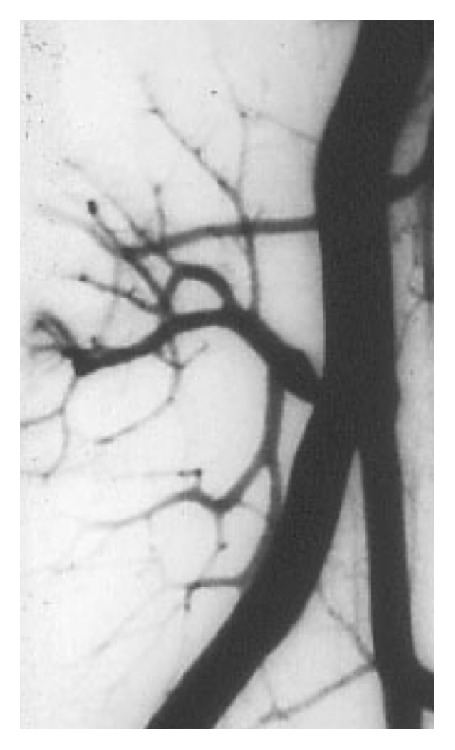
Arterial stenosis in a kidney transplant recipient (case 4).

The age difference between donor and recipient was ≤15 years in 87 patients (53.0%) (Group A) and >15 years in 77 patients (47.0%) (Group B, *p* = ns). No differences were found in terms of recipient age between groups or dialysis vintage, gender, causes of renal failure, hypertension, smoking, or hypercholesterolemia (Table 2). The number of HLA antigen mismatches was 2 ± 1 in Group A versus 1 ± 2 in group B (*p* = ns). A significant difference in the donor age was found between the groups: 40 ± 8 years in group A versus 22 ± 4 years in Group B (*p* < 0.05). Four renal arteriograms were carried out in Group A patients and 9 arteriograms were carried out in Group B. None of the Group A patients developed an anastomotic arterial stenosis, whereas 8/77 Group B patients (10.4%) had anastomotic arterial stenosis (Figures 5–9). The difference observed in the fraction of patients presenting arterial stenosis was statistically significant (*χ* ^2^ test = 5.51; *p* < 0.001). No difference in kidney function between groups was observed at diagnosis: creatinine was 1.4 ± 0.2 mg/dL in Group A versus 1.3 ± 0.3 mg/dL in Group B and the glomerular filtration rate was 85 ± 25 mL/min in Group A versus 88 ± 30 mL/min in Group B, *p* = ns. It is noteworthy that Group B patients presented renal arterial stenosis within six months of kidney transplantation.

## 6. Discussion

The incidence of renal artery stenosis after renal transplantation varies in the literature ranging from 1.0% to 23% [12, 19]. Arterial stenosis constitutes one of the main problems in transplant outcome since it can directly determine a reduction in graft perfusion and hence the development of both hypertension and atherosclerosis. This stenosis can lead to reduced blood perfusion up to blood flow arrest in this point of the vessel resulting in necrosis of the implanted organ [21–23]. In kidney transplantation, this problem may also lead to accelerated organ failure and the need for dialysis [23, 24]. Commonly the age difference between the donor and the recipient is a significant risk factor for graft survival but this topic is not assessed for the onset of arterial graft stenosis. Busson and Benoit considered a huge cohort of 6889 cadaver kidney grafts from 1 January 1989 to 31 December 1992 to analyze the impact on graft survival of matching for sex and age between donors and recipients. The results of the multivariate analysis showed that the main risk factors controlling graft survival are preimmunization before the graft, HLA-DR incompatibility, retransplantation, donor sex, and matching for age between donor and recipient [16]. Kostakis et al. assessed that the age difference between donor and recipient, with a cut-off point of 13 years, was the only statistical significant risk factor for long-term allograft survival after living donor renal transplantation in a caseload of 478 patients enrolled between 2000 and 2012 [17]. No mention was reported on renal artery stenosis and age difference between donor and recipient. Recent studies by Hagen and Ghazanfar focused on the kidney graft artery stenosis with percutaneous transluminal renal angioplasty but they did not assess the risk factors related to this disease [19, 20]. Ghazanfar et al. showed that the donor mean age was 42.6 ± 11.9 years and it did not differ from the recipient mean age that was 44.4 ± 11.4 years [20]. Colì et al. in 2006 focused on the mechanical properties of great arterial vessels after renal transplantation. Colì et al.'s paper assessed 92% of anastomotic arterial stenosis in 38 kidney transplant patients with age difference between donor and recipient >15 years. The incidence of arterial stenosis was 5.7% in 314 kidney transplant patients where the age difference was <15 years [25]. Nonetheless in this paper the arteriographic narrowing for the definition of a stenotic lesion was not shown, nor was the description of the characteristics of the patients and the criteria for donor and recipient selection for renal transplantation [25].

The present retrospective evaluation of a cohort of kidney-transplanted patients confirms the hypothesis that the difference in the mechanical properties of the donor/recipient arterial wall plays a role in the development of anastomotic artery stenosis in the graft. In our study the caseload reported by Colì et al. has been completely revised: we selected 164 patients with a complete case history among 352 patients who received kidney transplantation from 1981 to 1991. The results of the renal graft selective angiography were reevaluated: an arteriographic narrowing ≥50% was considered significant for arterial stenosis combined with the clinical history of the patients, as reported by Hagen and Ghazanfar [19, 20]. The results validate the mathematical relationship between wall impedance and blood flow disturbances [26, 27].

If the difference in mechanical impendence between the two arterial walls is negligible, nearly all the energy crosses the suture. If it is not negligible, the energy transmitted is smaller than that reflected. Blood flow loses its laminar characteristics and generates turbulent fluxes, which may create risk conditions for vascular complications [10]. An implanted organ is connected by a suture with one of its vessels and the circulatory system of the patient. The colloquium between the two parts, the receiving body and the foreign organ, is conducted by blood [28]. We are faced with two similar but not equal sutured walls; Young's modulus of elasticity will never be equal in the two. This point sometimes gives rise to mechanical rejection due to the different diameters of the two vessels connected. In particular, if the second vessel is softer than the first, it will soon generate a stenosis due to the turbulent movement of the blood in the initial part of the new artery and the process of blood cell sedimentation. Consequently, the point of suture must always be considered because it may present a point of discontinuity in the mechanical characteristics of the connected vessels. When the vessels are quite different, such as when their difference in age is >15 years, nonlaminar blood flow with the development of vortexes and turbulence may give rise to vessel lumen stenosis.

The main weakness of the study is follow-up that lasted only 12 months. The kidney graft arterial stenosis has not been recorded after this period due to the retrospective schedule of the study that did not allow to assess exactly the changes in the immunosuppressive therapies, the number of acute rejections, the onset of chronic allograft nephropathy, and the changes in eating habits or in cigarette smoking. Nonetheless in Colì et al.'s study 35 out of 38 patients developed an anastomotic arterial stenosis within 6 months from kidney transplantation and it can allow us to speculate that the mechanical difference between the great arteries of the donor and of the recipient is an early trigger for the anastomotic arterial stenosis after transplantation [25].

This mechanical phenomenon, related to the different age of donor and recipient in renal transplantation, is defined as the “bononiensis control parameter” [22].

In human pathology, vessels with different mechanical properties are connected not only in kidney transplantation but also in all organ transplants and whenever a prosthetic or human vascular segment is inserted in a patient's arterial vessel. As a result, the difference in the mechanical properties of connected vessels generates blood flow turbulence and shear stress when blood and wall biology systems are activated, with the risk of stenotic complications like those encountered in renal transplantation.

In conclusion, we analyzed the mechanical properties of the great arterial vessels and their influence on blood flow when connected to vessels with different mechanical characteristics. Our findings show that more consideration should be given to patients' age difference in organ and vessel transplantation and that more research is needed in new vascular prosthetic materials with mechanical properties more similar to human vessels.

## Figures and Tables

**Figure 9:**
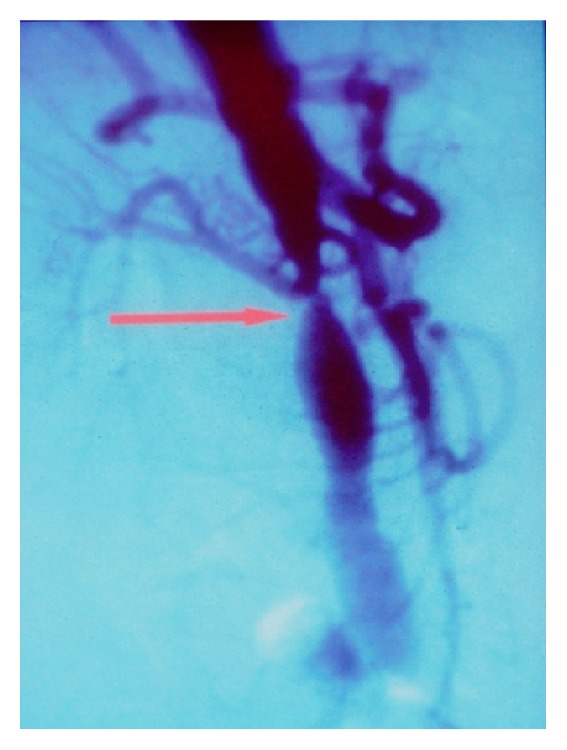
Arterial stenosis in a kidney transplant recipient (case 5).

**Table 1:** Values of the experimental velocities and attenuations.

Velocity	Attenuation
*V*_*r*_ = 11 m · s^−1^	*α*_*r*_^2^ = −1.1
*V*_*a*_ = 30 m · s^−1^	*α*_*a*_^2^ = −3.6
*V*_*t*_ = 18 m · s^−1^	*α*_*t*_^2^ = −4.3

**Table 2:** Characteristics of the patients.

	Group A	Group B	*p*
Patients (*n*)	87	77	
Age (years)	39 ± 11	41 ± 8	ns
Donor age (years)	40 ± 8	22 ± 4	*p* < 0.05
M/F	41/46	37/40	ns
Dialysis vintage (months)	19 ± 12	18 ± 15	ns
Hypertension (%)	46	52	ns
Smoking (%)	14	15	ns
Hypercholesterolemia (%)	22	23	ns
Causes of renal failure (%)			
(i) Glomerulonephritis	63	62	ns
(ii) Interstitial	17	19	ns
(iii) Hypertension	15	14	ns
(iv) Polycystic disease	5	5	ns
